# Proximal interphalangeal-level fracture in patient with symphalangism

**DOI:** 10.1080/23320885.2022.2123808

**Published:** 2022-09-28

**Authors:** Tommy Pan, Don Hoang, Alexander Payatakes

**Affiliations:** a Department of Orthopaedics, Allegheny General Hospital, Pittsburgh, PA, USA; b Penn State College of Medicine, Hershey, PA, USA; c Penn State Hershey Medical Center, Bone and Joint Institute, Department of Orthopaedics and Rehabilitation, Hershey, PA, USA

**Keywords:** Symphalangism, congenital, fracture, hand, genetics, ankylosis

## Abstract

Symphalangism is a rare, congenital syndrome involving ankylosis of the interphalangeal joints. We present a rare case of fracture at the level of a fused proximal interphalangeal joint in a patient with proximal symphalangism of the hand. Nonoperative management with splinting resulted in osseous healing and restored baseline function.

## Introduction

Symphalangism, described by Dr. Harvey Cushing in 1916, is a rare, hereditary syndrome involving the ankylosis of the interphalangeal (IP) joints of the fingers and/or toes [1–7]. The proximal interphalangeal (PIP) joint of the small finger is the most common site of ankylosis followed by the ring, middle and index fingers [8]. Compensatory hyperflexibility of unaffected joints often develops [9].

Many physicians may be unfamiliar with this condition and have difficulty interpreting its radiographic findings, especially given the various degree of ankylosis. Distinguishing the radiographic findings of symphalangism from degenerative changes or occult trauma may be challenging. Limited literature exists on the management of fractures associated with symphalangism. We present a rare case of fracture through a completely fused PIP joint in a patient with congenital proximal symphalangism of the hand.

Our goal is to increase hand surgeons’ and radiologists’ familiarity with this rare congenital condition and present our experience with nonoperative management of a fracture complicating this condition. We also review the available literature, including the underlying genetics and resultant biomechanics of this condition. Our hypothesis is that PIP fusion increases the mechanical lever arm of the applied force, potentially predisposing to fracture and adversely affecting subsequent healing.

## Case report

A 39-year-old, right-hand dominant Caucasian male was referred to hand clinic for evaluation of sequelae of an injury to his left ring finger, which he sustained 5 months prior to presentation. He was working as an automobile mechanic, when he ‘jammed’ his ring finger. He did not immediately seek medical attention, believing it to be a sprain. Over the next several weeks, he had persistent discomfort at baseline and pain while performing vigorous, manual labor. He initially presented to his rheumatologist four months after the initial injury. Radiographs revealed a cortical irregularity at the level of a congenitally fused left ring finger PIP joint. The patient was prescribed celecoxib and referred to hand clinic for further evaluation and management. He was a non-smoker.

The patient reported chronic stiffness of his fingers since childhood but was able to adjust and successfully perform manual labor as an automobile mechanic without significant disability. He had previously undergone a negative rheumatology workup for polyarthralgia. Multiple members of his family, which belonged to the Old Order Amish, had ‘stiffness’ and early-onset osteoarthritis spanning several generations.

Evaluation of the patient’s hands revealed that the PIP joints of his bilateral index through small fingers postured in extension with associated absence of corresponding dorsal skin creases. Bilateral small finger distal interphalangeal (DIP) level camptoclinodactyly was also noted. There was no active or passive flexion of the bilateral index through small finger PIP joints. Mild swelling and tenderness were present at the left ring finger PIP joint. He had moderate pain with attempted passive flexion of the joint, but no gross instability. The metacarpophalangeal (MCP) and DIP joints of the affected digits exhibited above average active and passive flexion. The remaining non-ankylosed joints were grossly normal in appearance and demonstrated no obvious degenerative changes. The patient had satisfactory overall function of his bilateral hands. He was able to strongly grasp objects with good compensation at the MCP and DIP levels. He was neurovascularly intact. Examination of lower extremities also revealed PIP level ankylosis of his toes bilaterally and mild pes planus. The patient demonstrated no signs of hearing loss.

**Figure 1.**
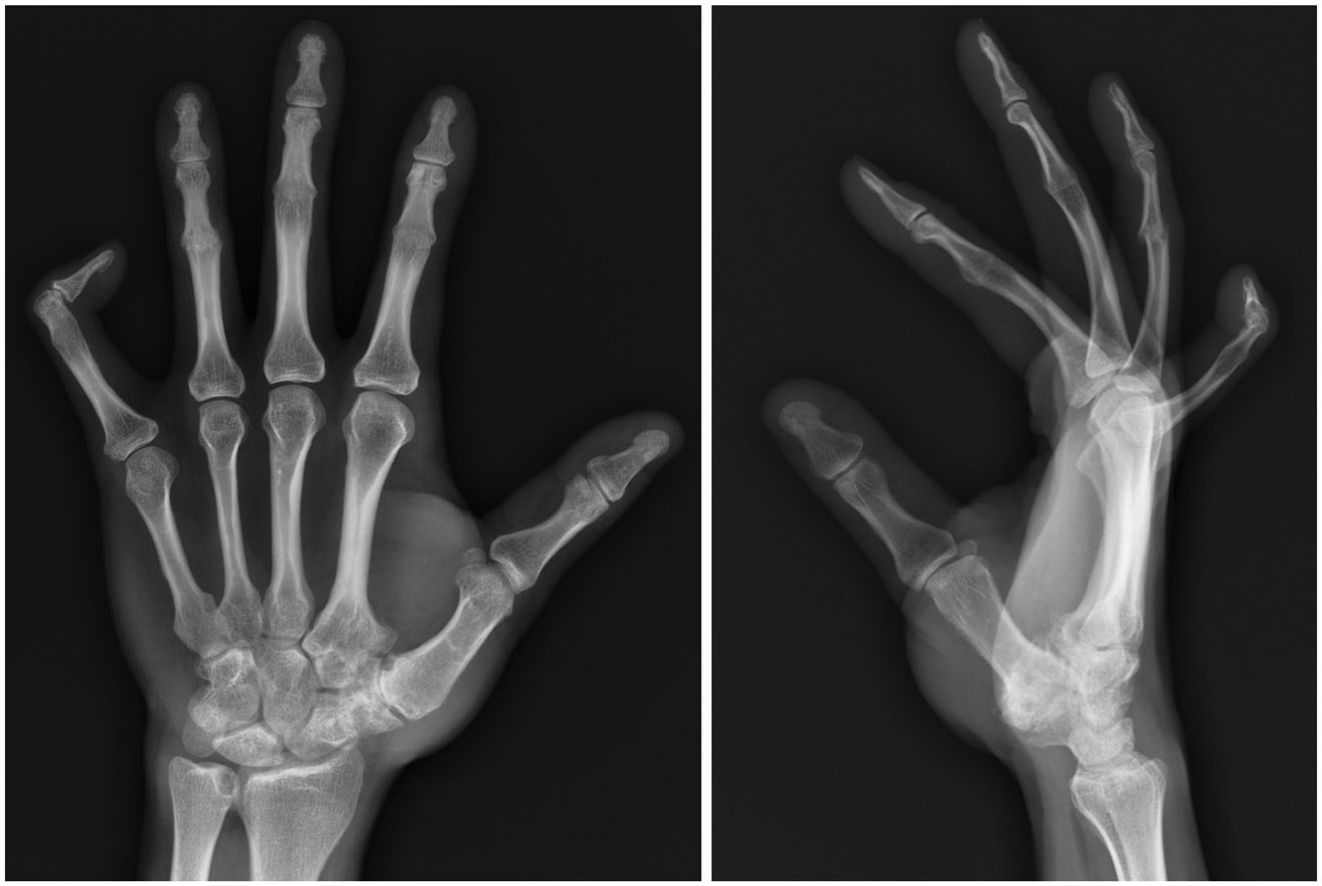
Plain radiographs of the left hand of a 39-year-old male of Amish descent with congenital proximal symphalangism demonstrating bony fusion of the second through fourth PIP joints and camptoclinodactyly of the fifth DIP joint.

**Figure 2.**
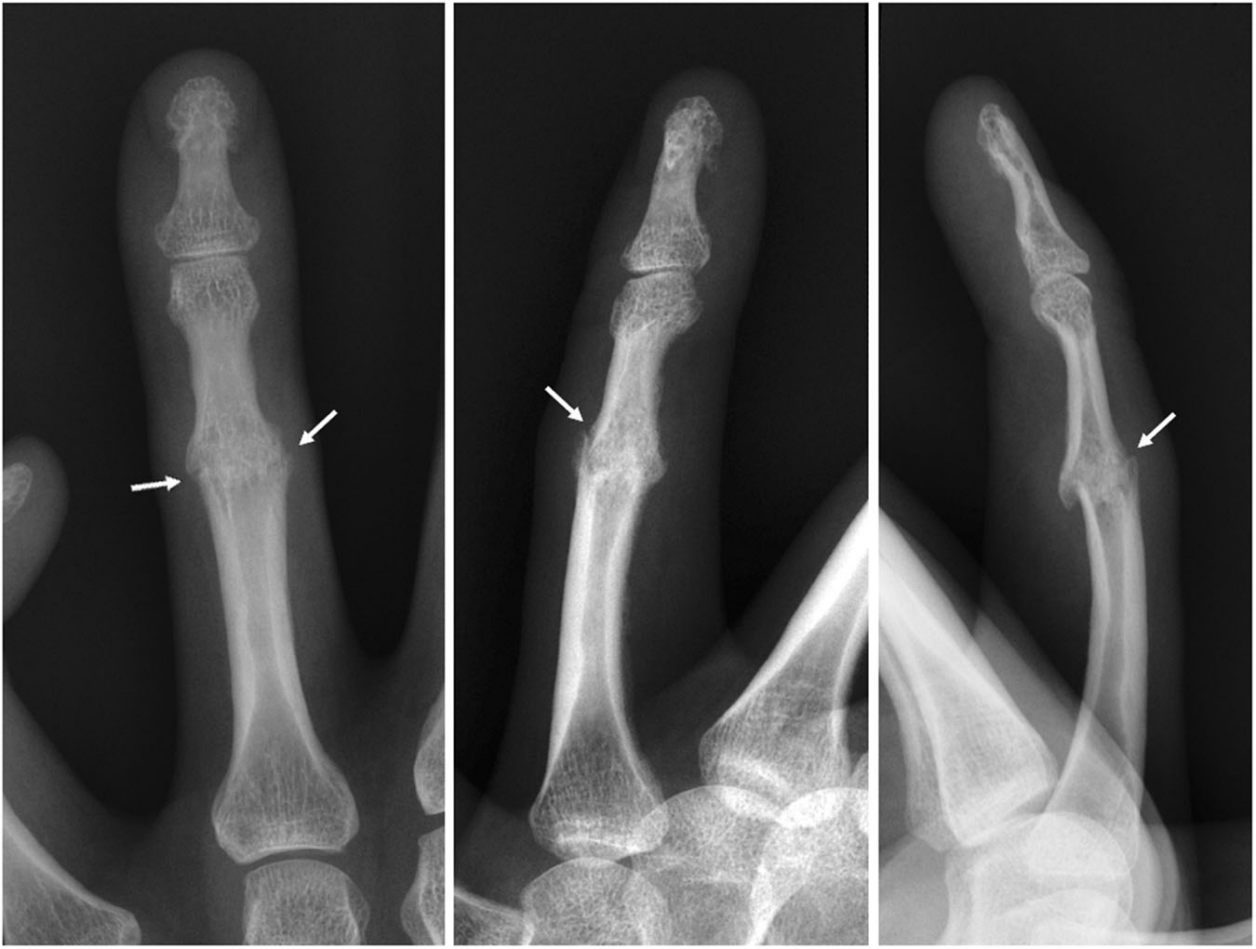
Plain radiographs of the left fourth digit reveal cortical irregularity at the level of the fused PIP joint (*arrows*) and adjacent soft tissue swelling.

Radiographs of his bilateral hands revealed complete bony ankylosis in extension of the PIP joints in his bilateral second through fourth digits (Figure 1). The DIP joint of the fifth digit postured in a hyperflexed position. Moderate degenerative changes of the thumb carpometacarpal (CMC) joints were noted, as well as mild osteoarthritic changes throughout the DIP joints. Radiographs of the affected left ring finger revealed persistent cortical irregularity in the region of the fused PIP joint with mild, adjacent soft tissue swelling, consistent with a subacute fracture (Figure 2).

**Figure 3.**
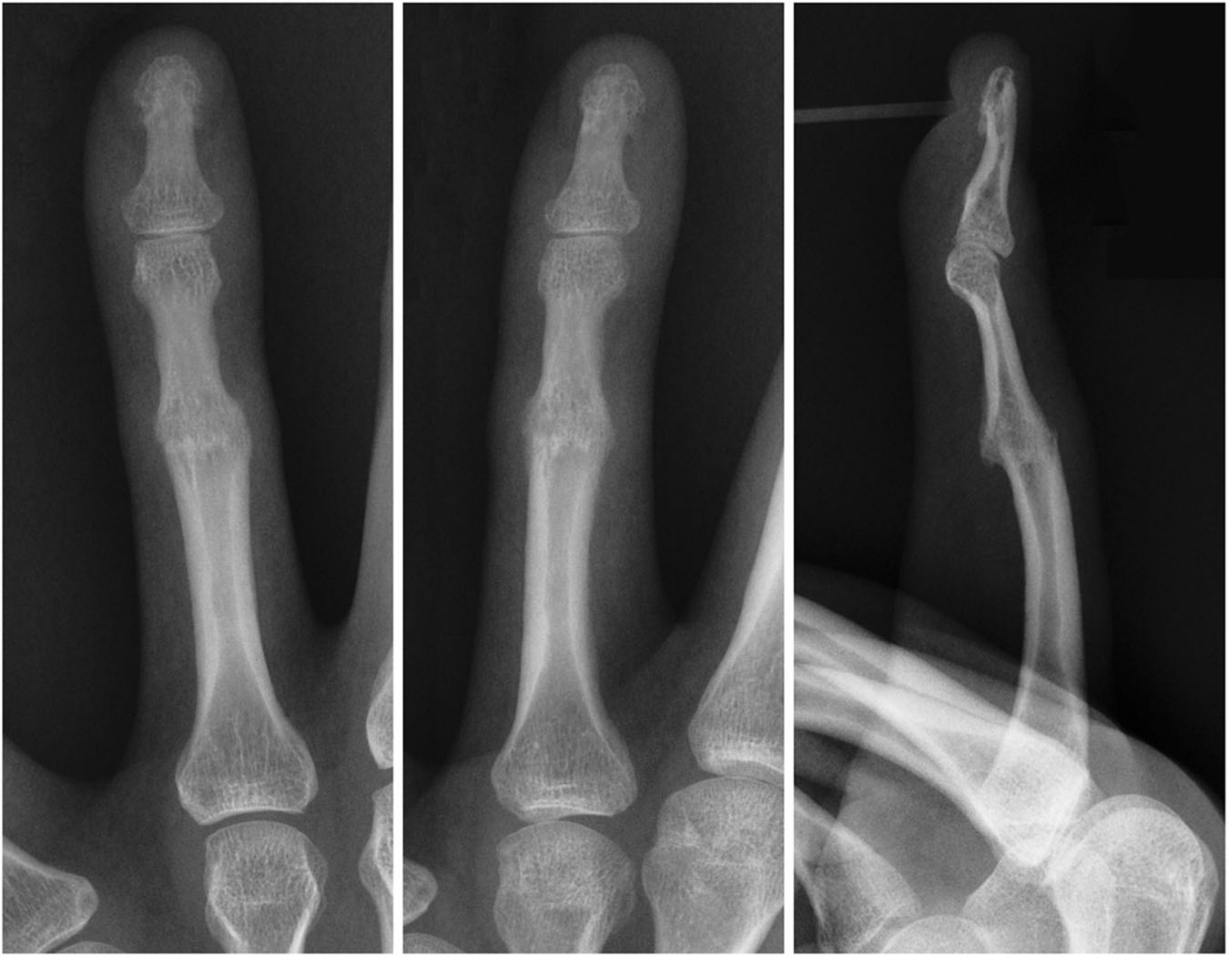
Plain radiographs of the left ring finger after 6 weeks immobilization demonstrating mature trabecular bridging across the PIP joint.

Clinical and radiographic evaluation were consistent with subacute fracture/delayed union complicating underlying bony proximal symphalangism (Grade III) (as opposed to incomplete fusion). The patient was treated with a custom thermoplastic volar finger-based splint immobilizing the PIP ‘joint’ in full extension, allowing motion at the MP and DIP levels. Discontinuation of the celecoxib was recommended. Radiographs at 3-weeks follow-up revealed persistent cortical irregularity but less prominent lucency and soft tissue swelling at the PIP level. At 6-weeks follow-up, the patient was pain-free. His ring finger was completely nontender and clinically stable with manipulation at the PIP level. Radiographs revealed mature trabecular bridging across the fracture with restoration of the congenital bony fusion (Figure 3). The splint was discontinued and the patient returned to work. At 1-year follow-up, the patient was successfully working. He was having ongoing generalized polyarthralgia, managed by his rheumatologist, and unrelated to his left ring finger injury.

## Discussion

Symphalangism is typically inherited in an autosomal dominant pattern. It has been classified into 3 types: (1) true symphalangism (involving digits with normal length), (2) symbrachydactylism (involving digits that are short and stiff), and (3) symphalangism with associated anomalies (e.g. Apert’s, Poland’s or multiple synostosis syndrome) [3,7,10].

**Figure 4.**
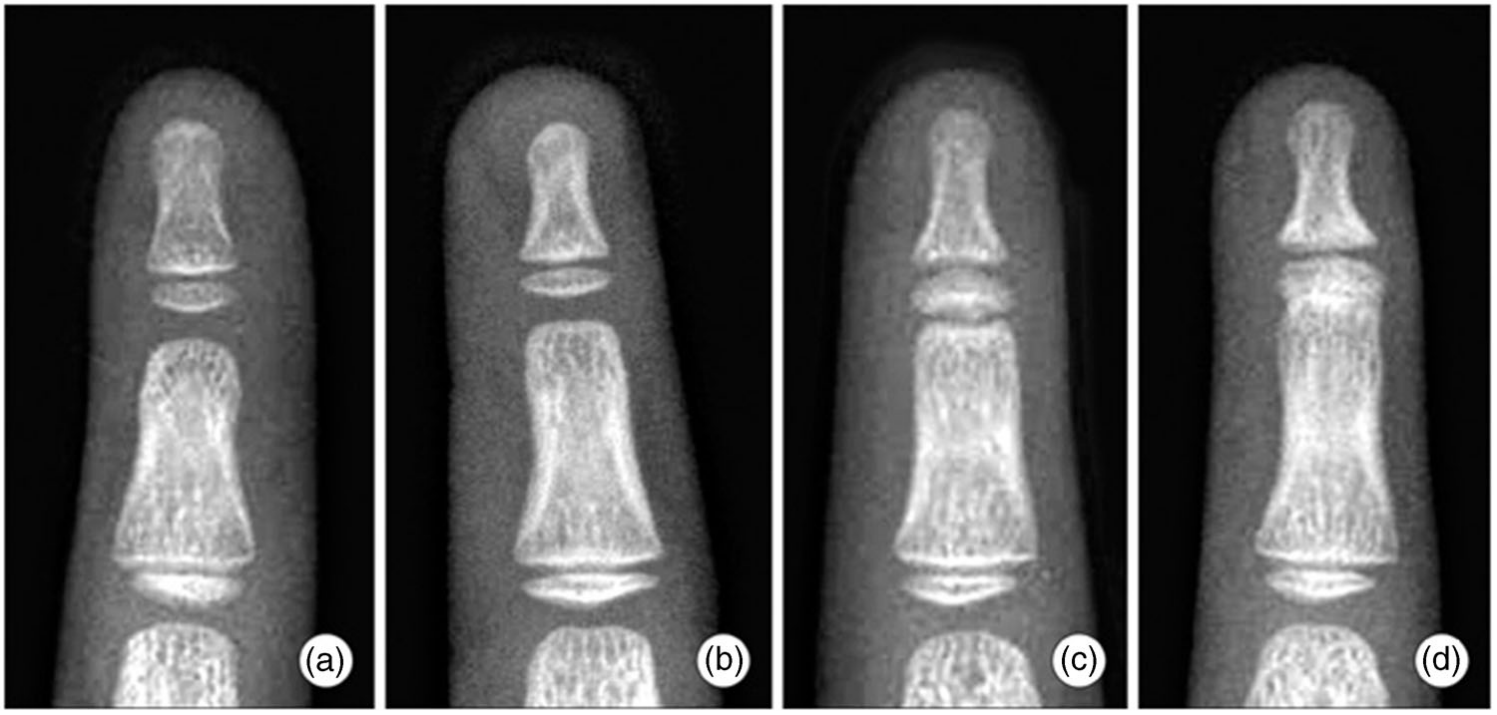
Grades of symphalangism: (A) Normal joint. (B) Grade I: fibrous symphalangism – mild joint space narrowing in distal interphalangeal joint. (C) Grade II: cartilaginous symphalangism – minimal joint space is observed. (D) Grade III: bony symphalangism. (reprinted from Baek, Lee [Baek 8]: https://www.ecios.org/DOIx.php?id=10.4055/cios.2012.4.1.58) under the Creative Commons Attribution Non-Commercial License: https://creativecommons.org/licenses/by-nc/3.0/)

Symphalangism may additionally be categorized into 3 grades, based on the degree of fusion (Figure 4) [Baek 8]. Fibrous symphalangism (Grade I) is characterized by mild joint narrowing, and approximately 10–20° of passive ROM. Cartilaginous symphalangism (Grade II) is characterized by minimal joint space and residual micromotion only. Bony symphalangism (Grade III) is characterized by osseous bridging of the joint with absence of micromotion. During childhood, progression from one grade to another is possible with increasing severity of ankylosis.

Symphalangism is often associated with other skeletal abnormalities such as camptodactyly, clinodactyly, syndactyly, radiohumeral fusion, pes planus, bilateral hip dislocation, tarsal coalition, and congenital fusion of the cervical or thoracic spine [10]. The fused phalanges result in diminished hand function with inability to make a fist and difficulty performing fine movements. Associated findings may include absence of cutaneous creases over the affected joints and conductive hearing loss [8].

Recent literature continues to elucidate the underlying genetic causes of proximal (PIP level) symphalangism (SYM1). SYM1 is typically an autosomal dominant condition most commonly associated with mutations in the Noggin (NOG) or Growth differentiation factor 5 (GDF5) genes [11–13]. Noggin is a bone morphogenic protein (BMP) antagonist that is essential to the regulation of multiple signal pathways and morphogenesis of cartilage, bone, and joints [14–16]. GDF5 is a growth agonist of the transforming growth factor-β (TGF-β) family involved in growth, repair and reconstruction of cartilage and bone. Mutations that result in the underexpression of NOG or overexpression of GDF5, can result in bony fusion [17,18]. NOG mutations are also associated with conductive hearing loss [19].

Management of congenital symphalangism often consists of benign observation, as patients typically adjust well to the limitations of this condition. In the pediatric population, occupational therapy (OT) may improve dexterity and even increase mobility in cases of symphalangism with residual motion. Surgical treatment of Grade I or II symphalangism has been reported, including joint release, dorsal Z-plasty and/or interposition arthroplasties [8,12,20]. These procedures have had high failure rates, often result in re-ankylosis and are not applicable to patients with open physes [21]. OT may be beneficial in adults, whereas surgical intervention is rarely indicated [10]. Surgical mobilization has yielded unsatisfactory functional results and poor patient satisfaction rates [22]. However, Palmieri reported silastic arthroplasty in 4 adult patients with Grade III (bony) proximal symphalangism with successful restoration of limited PIP mobility (average 50°arc) in non-border digits [23].

PIP joint symphalangism, with its various degrees of ankylosis, may potentially complicate interpretation of hand radiographs. Low-grade (non-bony) fusions may be misinterpreted as simple arthritic changes. Conversely, subtle secondary fractures may be hard to distinguish from higher grade fusions. While congenital symphalangism has been well described in the literature, we are aware of only been two prior reports of symphalangism complicated by fracture. Both failed conservative management. The first case was a 28-year-old gardener that presented with a 2-month history of index finger pain after suffering a direct blow. Radiographs revealed distal symphalangism of the index, middle, and ring fingers with a fracture through the DIP joint of the index. Immobilization in a stack splint for 8 weeks was unsuccessful in achieving union. Surgical intervention consisting of removal of residual cartilage, bone grafting, and K-wire fixation for 6 weeks was successful in achieving osseous union [24]. The second case was a 60-year-old female with DIP symphalangism of the 4th and 5th toes who sustained a fracture to the fourth distal phalanx when a trolley ran over her toe. Treatment with buddy splinting for 3 months with subsequent transition to short walking boot led to painful nonunion. Open reduction internal fixation with a headless compression screw resulted in radiographic osseous union 16 weeks later [25]. In contrast, our patient had proximal symphalangism with superimposed fracture at the PIP level. Extension splinting *in situ* achieved osseous union at 6-weeks despite delayed presentation. He was able to successfully return to his prior manual job and exhibited no complications or sequelae of his injury at 1-year follow-up.

It has long been established that other ankylosing disorders of the spine and hip predispose to fracture. The ankylosis creates a longer lever arm and therefore a larger moment [26–28]. This larger moment, in turn, increases stress as expressed in the equation for bending stress: *σ* = (Mc)/I, where σ is the bending stress, M is the bending moment, c is distance from the neutral axis, and I is the moment of inertia around the neutral axis [29]. These long lever arms can consequently predispose to fracture from even minor trauma [27,28]. In the case of symphalangism, it is reasonable to extrapolate that the fracture through the ankylosed PIP joint in our patient was, to some degree, the result of a longer lever arm due to the presence of one long, rigid macrophalanx (as opposed to multiple, smaller and more mobile phalanges). Relatively minor injury may have thus generated a higher bending moment and bending stress. Similar principles apply to surgical management of fractures in ankylotic disorders. The risk of failure of the surgical construct should be minimized by increasing the rigidity of the construct and extending it proximally/distally [28]. With regards to symphalangism, the applied moments are significantly lower, but it is reasonable to suggest that the increased bending stress may explain the failure of nonoperative management in the two prior case reports, as well as the delayed union of the minimally displaced fracture in our patient until adequate immobilization was achieved. Finally, it is known that joint fusions predispose adjacent joints to accelerated degeneration (e.g. adjacent segment disease). It has similarly been shown that symphalangism can lead to early osteoarthritis of the adjacent PIP/DIP joints due to increased mechanical stress [30]. Our patient was reporting generalized arthralgias in his bilateral hands at his most recent follow-up with his rheumatologist.

## Conclusion

We present a 39-year-old male with proximal (PIP) symphalangism complicated by fracture. Conservative management with splinting achieved bony healing at 6 weeks and excellent outcome, despite delayed presentation. Knowledge of the spectrum of severity and appreciation of the increased biomechanical stress may allow the hand surgeon to appropriately recognize the atypical findings on radiographs and properly manage this rare condition.

## Ethical approval

Our institution does NOT require ethical approval for reporting individual cases or case series.

## Informed consent

There is NO information (names, initials, hospital identification numbers or photographs) in the submitted manuscript that can be used to identify patients.

## References

[1] Cushing H. Hereditary ankylosis of proximal phalangeal joints (symphalangism). Genetics. 1916;1(1):90–106. 17245852 10.1093/genetics/1.1.90 PMC1193655

[2] Dellon AL, Gaylor R. Bilateral symphalangism of the index finger. J Bone Joint Surg Am. 1976;58(2):270–271. 1254638

[3] Flatt AE, Wood VE. Rigid digits or symphalangism. Hand. 1975;7(3):197–214. 173632 10.1016/0072-968x(75)90055-8

[4] Steinberg AG, Reynolds EL. Further data on symphalangism. J Hered. 1948;39(1):23–27. 18912544 10.1093/oxfordjournals.jhered.a105757

[5] Matthews S, Farnish S, Young ID. Distal symphalangism with involvement of the thumbs and great toes. Clin Genet. 1987;32(6):375–378. 3436086 10.1111/j.1399-0004.1987.tb03152.x

[6] Poush JR. Distal symphalangism. A report of two families. J Hered. 1991;82(3):233–238. 2061594 10.1093/oxfordjournals.jhered.a111071

[7] Borah D, Wadhwa S, Gupta AK, et al. Symphalangism in an Indian family. Ind J Phys Med Rehabil. 2006;17(1):18–20.

[8] Baek GH, Lee HJ. Classification and surgical treatment of symphalangism in interphalangeal joints of the hand. Clin Orthop Surg. 2012;4(1):58–65. 22379556 10.4055/cios.2012.4.1.58 PMC3288495

[9] Inman OL. Four generations of symphalangism. J Hered. 1924;15(8):329–334.

[10] Joshi A, Nagaraj C, Saurabh S, et al. Symphalangism-role of physical therapy. Eur J Radiol Extra. 2008;65(3):101–103.

[11] Polymeropoulos MH, Poush J, Rubenstein JR, et al. Localization of the gene (SYM1) for proximal symphalangism to human chromosome 17q21–q22. Genomics. 1995;27(2):225–229. 7557985 10.1006/geno.1995.1035

[12] Nightingale C, Kyprianou K, Syed M. A strongly inherited familial symphalangism of all the proximal interphalangeal joints. Plastic Surgery Case Studies. 2020;6:2513826X2097035–2513826X2097033.

[13] Baek GH, Kim J, Park JW. Mobilization of joints of the hand with symphalangism. Hand Clin. 2017;33(3):551–560. 28673631 10.1016/j.hcl.2017.04.008

[14] Potti TA, Petty EM, Lesperance MM. A comprehensive review of reported heritable noggin‐associated syndromes and proposed clinical utility of one broadly inclusive diagnostic term: NOG‐related‐symphalangism spectrum disorder (NOG‐SSD). Hum Mutat. 2011;32(8):877–886. 21538686 10.1002/humu.21515

[15] Marcelino J, Sciortino CM, Romero MF, et al. Human disease-causing NOG missense mutations: effects on noggin secretion, dimer formation, and bone morphogenetic protein binding. Proc Natl Acad Sci USA. 2001;98(20):11353–11358. 11562478 10.1073/pnas.201367598 PMC58733

[16] Gong Y, Krakow D, Marcelino J, et al. Heterozygous mutations in the gene encoding noggin affect human joint morphogenesis. Nat Genet. 1999;21(3):302–304. 10080184 10.1038/6821

[17] Plett SK, Berdon WE, Cowles RA, et al. Cushing proximal symphalangism and the NOG and GDF5 genes. Pediatr Radiol. 2008;38(2):209–215. 17994231 10.1007/s00247-007-0675-y

[18] Leonidou A, Irving M, Holden S, et al. Recurrent missense mutation of GDF5 (p.R438L) causes proximal symphalangism in a British family. World J Orthop. 2016;7(12):839–842. 28032038 10.5312/wjo.v7.i12.839 PMC5155261

[19] Sha Y, Ma D, Zhang N, et al. Novel NOG (p. P42S) mutation causes proximal symphalangism in a four-generation Chinese family. BMC Med Genet. 2019;20(1):133. 31370824 10.1186/s12881-019-0864-1 PMC6670124

[20] Dobyns J. Symphalangism. In: Green DP, Hotchkiss RN, Pederson WC, editors. Green’s operative hand surgery, 4th ed. New York: Churchill Livingstone; 1999. p. 470–473.

[21] Shibata M. Symphalangism. In: Gupta A, Kay SP, Scheker LR, editors. The growing hand. 1st ed. London: Mosby; 2000. p. 289–292.

[22] Durmus O, Cakar E, Ata E, et al. Symphalangism: ankylosis of the interphalangeal joints. Am J Phys Med Rehabil. 2014;93(1):90–91. 22660373 10.1097/PHM.0b013e31825a1695

[23] Palmieri TJ. The use of silicone rubber implant arthroplasty in treatment of true symphalangism. J Hand Surg Am. 1980;5(3):242–244. 7400561 10.1016/s0363-5023(80)80008-6

[24] Fortems Y, Smet LD, Stanley JK. Fracture with nonunion through an incomplete preaxial distal symphalangism. J Hand Surg Br. 1994;19(3):371–372. 8077831 10.1016/0266-7681(94)90092-2

[25] Foo GL, Wee LHJ. Surgical fixation and inter-phalangeal arthrodesis of symptomatic non-union of fracture of a lesser toe distal phalanx: a case report. Malays Orthop J. 2019;13(3):69–71. 31890114 10.5704/MOJ.1911.012 PMC6915320

[26] Kuroki H, Higa K, Chosa E. Clinical results of vertebral fracture related to diffuse idiopathic skeletal hyperostosis (DISH) which underwent conservative treatment: three case reports. Int J Spine Surg. 2021;15(1):195–202. 33900974 10.14444/8025 PMC7931743

[27] Furukawa M, Okuyama K, Kawano Y, et al. Femur bone mineral density and pentosidine level distinguish ankylosing spinal disorder patients with and without sacroiliac ankylosis. Spine Surg Relat Res. 2020;4(4):333–340. 33195858 10.22603/ssrr.2020-0001 PMC7661031

[28] Pato T, Malheiro FS, Estrela D, et al. Intertrochanteric fracture in a long-term ankylosed hip: a case report. J Orthop Case Rep. 2020;10(1):8–10. 32547969 10.13107/jocr.2020.v10.i01.1614 PMC7276569

[29] McKeen LW. Introduction to fatigue and tribology of plastics and elastomers. In McKeen LW ed. Fatigue and tribological properties of plastics and elastomers, 2nd ed., Vol. 1. Norwich: William Andrew Publishing, 2010.p. 1–23.

[30] Krohn KD, Brandt KD, Braunstein E, et al. Hereditary symphalangism. Association with osteoarthritis. J Rheumatol. 1989;16(7):977–982. 2769669

